# Using a PCR‐Based Method To Analyze and Model Large, Heterogeneous Populations of DNA†

**DOI:** 10.1002/cbic.201900603

**Published:** 2020-01-07

**Authors:** Helena Andrade, Alvin K. Thomas, Weilin Lin, Francesco V. Reddavide, Yixin Zhang

**Affiliations:** ^1^ B CUBE Center for Molecular Bioengineering Technische Universität Dresden Tatzberg 41 01307 Dresden Germany; ^2^ DyNAbind GmbH Arnoldstraße 18D 01307 Dresden Germany

**Keywords:** DNA, DNA-encoded chemical libraries, fluorescence, polymerase chain reaction, population modeling

## Abstract

The study of populations of large size and high diversity is limited by the capability of collecting data. Moreover, for a pool of individuals, each associated with a unique characteristic feature, as the pool size grows, the possible interactions increase exponentially and quickly go beyond the limit of computation and experimental studies. Herein, the design of DNA libraries with various diversity is reported. By using a facile analytical method based on real‐time PCR, the diversity of a pool of DNA can be evaluated to allow extraordinarily high heterogenicity (e.g., >1 trillion). It is demonstrated that these DNA libraries can be used to model heterogeneous populations; these libraries exhibit functions such as self‐protection, suitability for biased expansion, and the possibility to evolve into amorphous structures. The method has shown the remarkable power of parallel computing with DNA, since it can resemble an analogue computer and be applied in selection‐based biotechnology methods, such as DNA‐encoded chemical libraries. As a chemical approach to solve problems traditionally for genetic and statistical analysis, the method provides a quick and cost‐efficient evaluation of library diversity for intermediate steps through a selection process.

## Graphical abstract

**Uncovering diversity data**: The study of populations in large and highly diverse libraries is limited by the capability of collecting and analyzing data. A chemical method based on real‐time PCR can be used as a function (*F*) to calculate the diversity (*D*) and concentration (*C*) of a DNA library, which can be considered as a group of variables (*X*).

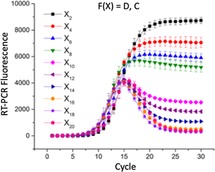

Large and dynamic populations of high diversity are difficult to sample, analyze, and model.^1^, ^2^ We propose that a synthetic DNA library represents the ideal medium for establishing experimental systems for modeling large populations of high complexity and dynamics. DNA libraries can be synthesized with controlled varying diversity; manipulated enzymatically; subjected to growth through PCR; and analyzed through various methods, including real‐time PCR (RT‐PCR or qPCR)^3^, ^4^ and sequencing.^5^ For example, a randomized 20‐base sequence (N_20_) contains more than one trillion different DNA sequences (4^20^). A sample (10 μL) of a 1 nm solution of N_20_ has the size of the human population, and statistically each molecule represents a unique individual. Although next‐generation sequencing has revolutionized the field of genomics,^6^, ^7^ the method cannot provide a quick evaluation of library diversity and is still limited to analyzing millions of different sequences.^8^, ^9^

Without performing a sequencing analysis, the difference between two double‐stranded DNAs can be evaluated by the probability of two sequences forming mismatched pairs with each other in a melting–reannealing process, and the melting temperature of the resulting “wrong” pairs. Although the principle has been used to design molecular beacons to detect mismatches, it can also be applied to access the heterogeneity of DNA libraries. For example, the effect of changing DNA population through SELEX cycles on RT‐PCR amplification and melting curves has been used to monitor the selection process.^10^ Herein, we have designed DNA libraries of various diversities. This system can be operated biochemically as an analogue computer with eventually unlimited number of variables as inputs. It can be used to model heterogeneous populations that exhibit functions such as self‐protection, suitability for biased expansion, and the possibility of evolving into amorphous structures. Our proposed method can be applied in selection‐based biotechnology methods, such as DNA‐encoded chemical libraries (DECLs).

The design of DNA libraries to model large populations of various diversities is shown in Figure 1 A. The 20‐base sequences, X_*n*_ (*n* is the length of the degenerate sequence and reflects the library diversity), are flanked by primers A and B. We first carried out a simulation of the PCR amplification process on libraries of different diversities (Figure S1 in the Supporting Information). In a high‐diversity library, two fully complimentary sequences have an extremely low probability of encountering each other during a melting–reannealing process, and thus, generating probabilistically self‐assembled mismatching pairs. We assume that each A‐X‐B sequence has the same probability of assembling with any A′‐X′‐B′ sequence, as well as with primer B′, whereas each A′‐X′‐B′ sequence has the same probability of forming a duplex with either A‐X‐B or A. The concentrations of A‐X‐B and A′‐X′‐B′ gradually increase through the PCR cycles, whereas those of primers A and B decrease.

**Figure 1:**
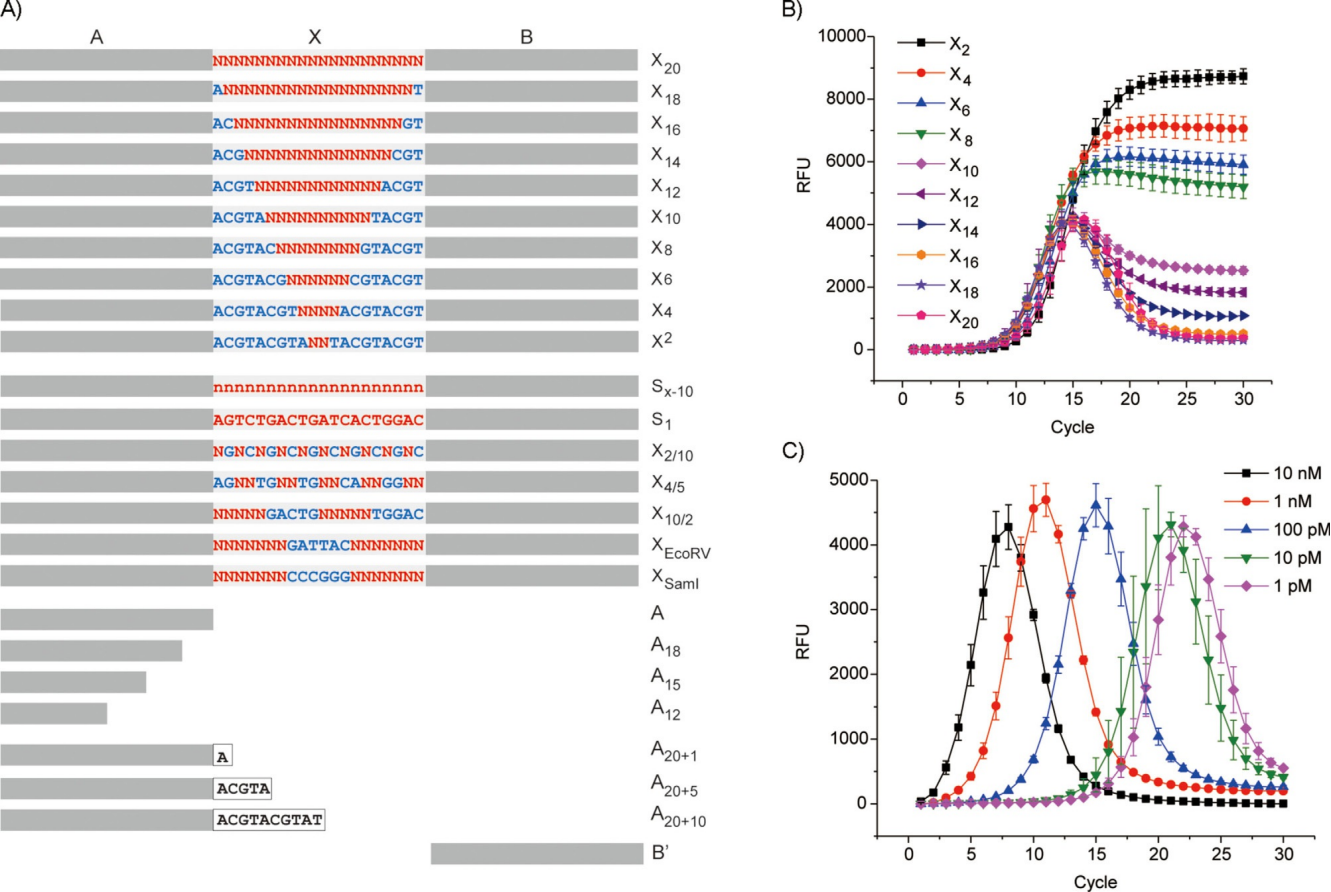
A) Design of DNA libraries and primers (Table S1). B) RT‐PCR experiments with 100 pm DNA of different diversities (from 2 to 20 random positions) as templates. C) RT‐PCR experiments with different concentrations of A‐X_20_‐B as a template.

We assigned a fully complimentary duplex with a functional factor (*F*) of 1.0. Mismatching leads to lower *F* values, for example, A‐*X_10_*‐B/A′‐*X_10_′*‐B′ and A‐*X_20_*‐B/A′‐*X_20_′*‐B′ duplexes have *F* values of 0.5 and 0.1, respectively (italic *X* and *X′* indicate mismatching in the randomized region). Interestingly, through the PCR cycles, although the production of full‐length DNA was not affected by using either a single DNA sequence, A‐S_1_‐B, or various libraries as templates, the time courses of *F* values differed from each other dramatically. In the initial phase, primer concentrations are much higher than those of full‐length DNA; probabilistically self‐assembled A‐X‐B/B′ and A′‐X′‐B′/A are more abundant than that of A‐*X*‐B/A′‐*X′*‐B′. A‐X‐B/B′ and A′‐X′‐B′/A produce A‐X‐B/A′‐X′‐B′ in the presence of DNA polymerase (underlined X/X′ indicates the fully complimentary pair synthesized by DNA polymerase, according to the template). The newly synthesized fully complimentary A‐X‐B/A′‐X′‐B′ possesses an *F* value of 1.0. In the later phase, the primers are consumed and full‐length DNAs are abundant. Hence, A‐X‐B/B′ and A′‐X′‐B′/A are much less prevalent than that of A‐*X*‐B/A′‐*X′*‐B′. The mismatching pairs have lower *F* values, in contrast to the duplexes produced by polymerase, for which *F*=1.0. There is a turning point in the course of a high‐diversity library (Figure S1), as the increase of *F* caused by newly synthesized fully complementary duplexes equals the decrease of *F* caused by the reannealing of DNA, which generates mismatched pairs through reshuffling between library members.

To relate the *F* value to an experimentally measurable parameter, we took advantage of the fact that the duplexes with more mismatches were less stable at elongation temperature; thus leading to weaker dye binding, and therefore, lower fluorescence. We first analyzed the effect of mismatching on the binding of fluorescence dye. A remarkably diminished fluorescence signal was detected if the mismatching number was ≥10, with a further decrease for ≥14 (Figure S1). Therefore, with the same amount of DNA duplexes, the fluorescence intensity reflects sequence mismatching, within the course monitored by RT‐PCR.^10^ It is important to note that a standard RT‐PCR protocol uses high primer concentrations to ensure the robustness of the assay. In our experiments, we adjusted primer concentrations to the range at which the primer concentrations exhibited a linear correlation with the final signal intensity (Figure S2).

As shown in Figure 1 B, DNA libraries of different diversities have shown RT‐PCR time courses that resemble the simulated curves. The low‐diversity library A‐X_2_‐B contains only 16 different species and shows a RT‐PCR profile similar to that of the sample containing only one sequence. RT‐PCR of the highest diversity library, A‐X_20_‐B, exhibited a peak‐shaped curve. With increasing diversity, standard RT‐PCR curves gradually transformed into peak‐shaped curves. Interestingly, although the curves of high‐diversity libraries are remarkably different from those in classical RT‐PCR measurements, the shifts of curves can be correlated with the template concentration (Figures 1 C and S3). Therefore, RT‐PCR can be used to determine the sample concentration and to illustrate sample diversity.

The difference in RT‐PCR curves between X_8_ and X_10_ is the most remarkable. However, the difference among X_4_, X_6_, and X_8_ or among X_10_, X_12_, and X_14_ is relatively small, whereas X_16_, X_18_, and X_20_ cannot be distinguished from each other. The difference can be augmented through tuning the elongation temperature. The difference among X_2_, X_4_, X_6_, and X_8_ has been drastically increased at 74 °C, whereas X_20_, X_18_, X_16_, and X_14_ can be clearly distinguished from each other at 68 °C (Figure 2 A, B). Therefore, besides covering a wide range of diversity, the conditions can also be tuned to focus on a relatively narrow range.

**Figure 2:**
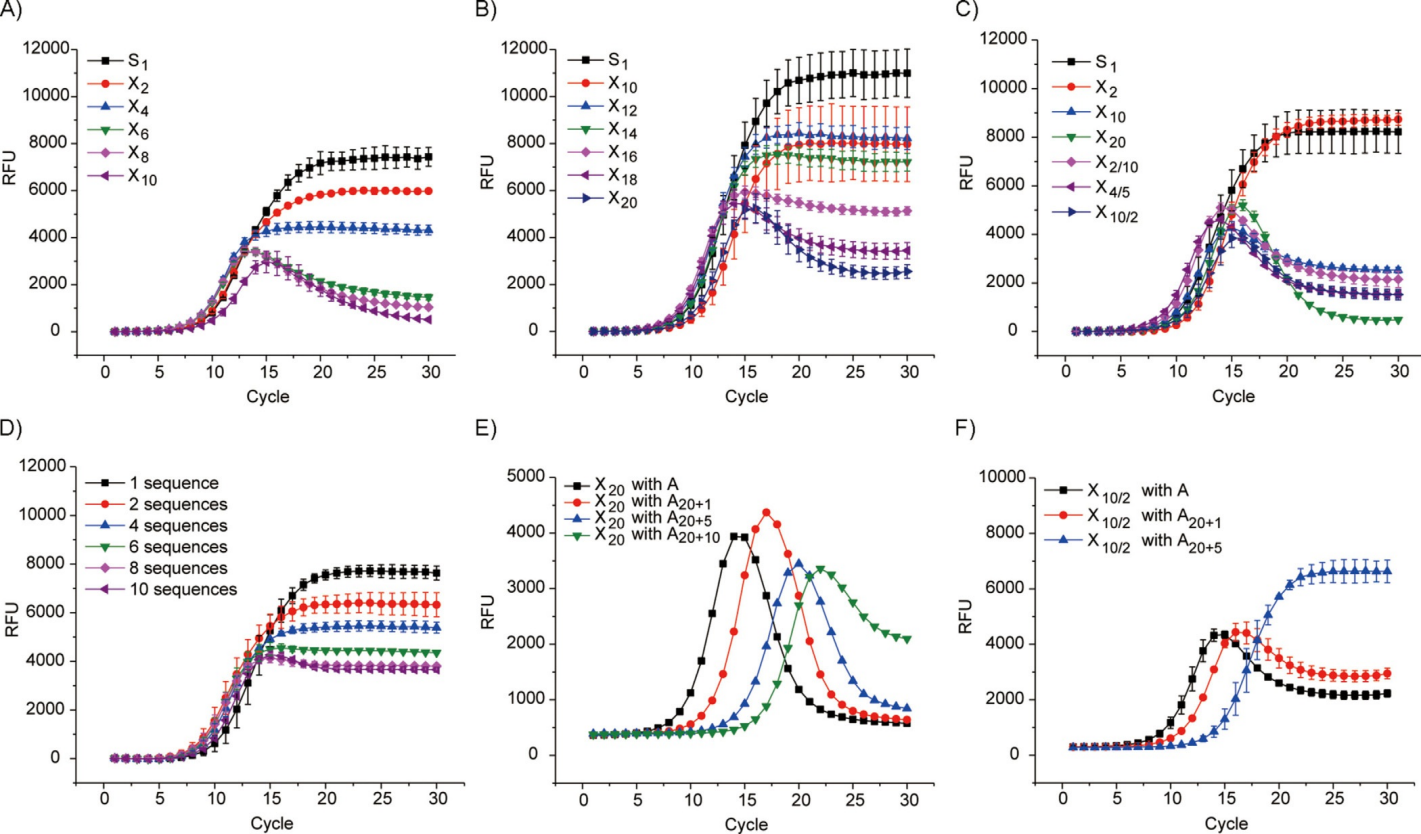
RT‐PCR amplification with elongation at A) 74 and B) 68 °C. C) RT‐PCR experiments on different libraries of the same diversity. X_10_, X_10/2_, X_2/10_, and X_4/5_ possess the same diversity, but different distributions of partially degenerate segments in the sequence. D) RT‐PCR experiments with a small number of different sequences. The sequences have been designed to possess a large difference (>15) between any pair. Manipulation of the population growth with A, A_20+1_, A_20+5_, or A_20+10_ as primers with the libraries E) X_20_ or F) X_10/2_. A_20+1_, A_20+5_, and A_20+10_ are primers of 21, 25, and 30 nt, respectively (Table S1). They can amplify only part of a partially degenerated library.

We then tested different libraries of the same diversity (Figures 1 A and 2 C). Although the partially degenerate part(s) are positioned very differently in the four libraries (X_10_, X_2/10_, X_4/5_, X_10/2_), the RT‐PCR experiments resulted in similar profiles. For library X_*n*_, if *n*>16, each sequence in a 1 nm sample (10 μL; e.g., X_18_ and X_20_) has statistically only a single copy, whereas each sequence in the X_2/10_ and X_4/5_ samples is presented a few thousand times. Interestingly, although X_20_, X_18_, X_16_, X_2/10_, and X_4/5_ possess randomized regions of similar length (18 to 20), the high‐diversity libraries (X_20_ and X_18_, Figure 1 B) produced curves clearly distinct from that of the medium‐diversity libraries (X_2/10_ and X_4/5_).

The difference between two sequences in the X_20_ library can be of any number between 0 and 20. We then designed a library of another type of diversity: a library containing *n* sublibraries. In this library, all members in one sublibrary are identical, but the difference between sublibraries is very high. We generated library S_*x*−10_ with ten sequences (Table S1), which were designed to ensure the difference between any two sequences was >15. If the annealing steps in the PCR cycles are too short for the formation of thermodynamically favorable complementary DNA duplex, RT‐PCR of S_*x*−10_ should produce a curve resembling that of X_20_. Interestingly, the resulting curve (Figures 2 D and S4) indicates that thermodynamic re‐equilibration does play a role in this process, although it is far too inefficient to cause perfect matching among all sequences. Reducing the sublibrary number transformed the curve to a standard RT‐PCR curve gradually.

We established a system in which RT‐PCR analysis could be considered as a “function”, with different DNA sequences in a mixture as input, and their diversity and concentration as output. We then tested whether the library diversity could be manipulated by varying the PCR primers and simultaneously analyzed by this function. A_20+1_, A_20+5_, and A_20+10_ (Figure 1 A) should selectively anneal to and amplify 1/4, 1/4^5^, and 1/4^10^ of the X_20_ library, respectively, and result in products of reduced diversity. As shown in Figure 2 E, the use of A_20+1_ caused a right shift of the RT‐PCR profile, whereas A_20+5_ caused a further shift. The diversity of both PCR products remains high. Curiously, if A_20+10_ was used, in addition to a further right shift of the curve, the shape transformed and resembled that of the X_10_ library; this is in good agreement with the reduced diversity of PCR product from 4^20^ (a X_20_ library) to 4^10^ (a X_10_ library). If X_10/2_ was subjected to RT‐PCR with A_20+1_ and A_20+5_ as primers, the diversities of products were reduced from 4^10^ to 4^9^ and 4^5^, respectively. The difference caused by biased amplification could be observed in both a right shift and shape transformation (Figure 2 F); this indicated a decreased number of templates and diversity. Because A_20_, A_20+1_, and A_20+5_ are fully complementary to X_10_, the primers did not affect the amplification curves, whereas they had a different effect on X_18_ (Figure S5).

To relate heterogeneity to a biochemical function, we designed the libraries A‐X_EcoRV_‐B and A‐X_SmaI_‐B. In X_EcoRV_ and X_SmaI_, two sequences for the restriction enzymes EcoRV and SmaI were inserted into the randomized regions, respectively (Figures 1 A and 3 A). This provides a mechanism of selection during growth (by PCR amplification) because the fully complimentary duplexes are optimal substrates for endonucleases (Figure 3 A). The mixture of A‐X_EcoRV_‐B and A‐X_SmaI_‐B was subjected to 5 or 25 cycles of PCR. Then, an aliquot of PCR product was treated with one of the restriction enzymes or both. After five cycles, most DNA duplexes were newly synthesized and fully complimentary. Enzyme treatments reduced their concentrations, especially in the presence of both enzymes (Figure 3 B). In contrast, after 25 cycles, the effect of enzyme treatment was abolished because the four curves could not be distinguished from each other (Figure 3 C). The emerging complexity caused by DNA reshuffling led to a design of heterogeneous population, which exhibited a protective effect against endonuclease digestion. In Figure 3 C, the surprising increase in fluorescence after 20 RT‐PCR cycles was further investigated.

**Figure 3:**
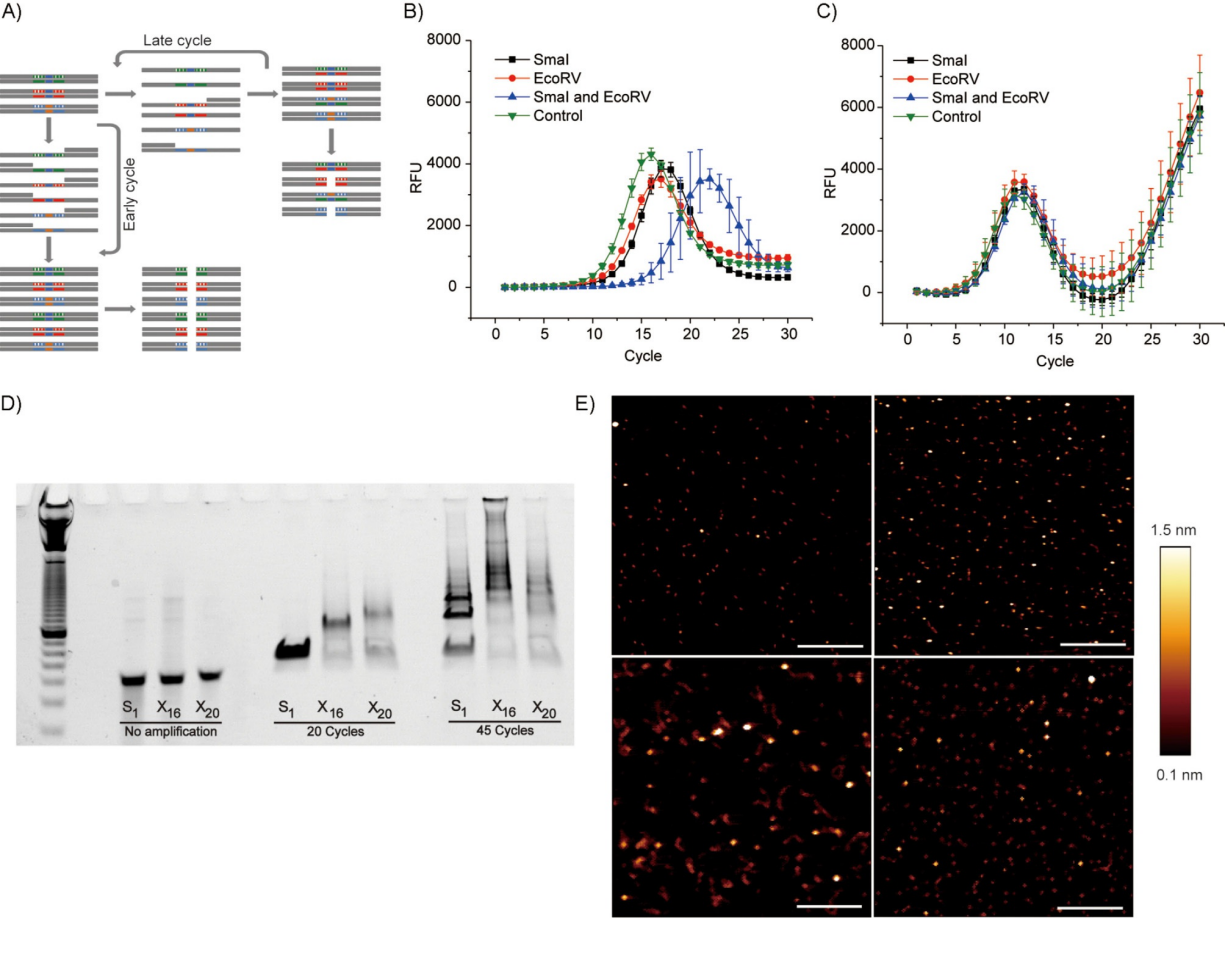
Diversity of libraries with restriction sites has a protective effect against endonuclease action. A) The fully complimentary duplex synthesized in the initial PCR cycles is more sensitive to endonucleases than that of the mismatched duplex generated in late PCR cycles. The mixture of A‐X_EcoRV_‐B and A‐X_SmaI_‐B was pretreated with B) 5 and C) 25 PCR cycles. A small aliquot of PCR product was treated with EcoRV, SmaI, or both and the products were subjected to RT‐PCR experiments. D) Singled‐stranded DNA samples of different diversity (S_1_, X_16_, X_20_) were subjected to 20 or 45 PCR cycles and separated by gel electrophoresis. The PCR‐amplified samples are double‐stranded. E) AFM analyses of PCR products of S_1_ after 20 (upper left) or 45 cycles (upper right), X_16_ after 45 cycles (lower left), and X_20_ after 45 cycles of amplification (lower right). Images are 1 μm×1 μm; scale bar: 250 nm.

A mixture of diverse sequences can cause many potential interactions and lead to structures other than fully complementary duplex and those involving more than two strands. A large library, such as A‐X_20_‐B, could involve interactions beyond the power of computation. We speculated that, in the presence of DNA polymerase, a highly diverse DNA library was more likely to grow into complex structures than that of a simple system.^11^ The first indication was an astonishing increase in fluorescence during the course of RT‐PCR if a degenerate library was pretreated with 25 PCR cycles (Figure 3 C). Without PCR pretreatment, an increase in fluorescence was observed only after more than 37 RT‐PCR cycles. We reasoned that, whereas the decrease in fluorescence was caused by the generation of less stable mismatching duplexes with low dye binding affinity at elongation temperature, the reoccurrence of fluorescence was caused by the formation of large and complex DNA structures, which were stabilized by an interwoven network.

To illustrate the formation of large DNA structures, single DNA sequence or libraries were subjected to 20 or 45 PCR cycles, and the products were analyzed by PAGE under denaturing conditions (Figure 3 D). After 20 cycles, bands corresponding to DNA larger than 60 bp were observed for the X_16_ and X_20_ samples, but not for the sample containing only one sequence (S_1_). After 45 cycles, high‐molecular‐weight bands appeared in all samples, whereas S_1_ still produced a strong 60 bp band. Interestingly, the X_16_ sample produced the largest shift. We then analyzed the PCR products through AFM.^12^ After 20 PCR cycles, mainly small structures of DNA duplex were observed for both S_1_ and X_20_ (Figures 3 E and S6). Many large structures could be detected after 45 PCR cycles for samples of X_12_, X_14_, X_16_, and X_20_, but not that of S_1_. The heights of single‐ and double‐stranded DNA chains measured by means of AFM are 0.3 and 0.7 nm, respectively, whereas here we observe hybrid multiplexes.^13^, ^14^ Structures showing heights remarkably greater than that of normal DNA duplex and genomic DNA have been observed, especially for the sample with X_16_ as a template.^15^ Because the generation of an amorphous 3D structure is associated with a highly diverse DNA library as a precursor, we called them high‐entropy structures, in contrast to self‐assembled DNA nanostructures with defined interaction networks, such as that in DNA origami.^16^

To demonstrate that the method can be used not only to build models, but also as a practical tool for existing library technologies, we applied the method to a DECL. One major challenge for a DECL is to find optimal selection stringency to discover small number of potent binders from large combinatorial libraries,^17^ by overcoming the low signal‐to‐noise ratio caused by promiscuous and/or weak interactions between DNA conjugates and target proteins immobilized on a solid support.^18^ Unfortunately, a selection experiment will remain a black box until the hit compounds are revealed after cumbersome steps of decoding and data analysis. This RT‐PCR method could lead to instant assessment of library diversity before and after selection, if the DNA codes are designed to create high heterogeneity.

We generated four model libraries using the code design of S_*x*−10_ (Figures 1 A and 2 D), in which the difference between any two sequences was >15. The library members were annealed with a 20‐mer (B′) modified with biotin (a high‐affinity binder to the protein streptavidin (SA)) or iminobiotin (a weak binder), or not modified (Figure 4 A). Lib‐A represents a library with a few potent binders without weak binders, and Lib‐B simulates a library containing a few weak binders. Lib‐C possesses many weak binders, whereas Lib‐D is used to mimic a library with a few high‐affinity ligands and many weak binders. The libraries were incubated with a SA‐coated resin. After removing the supernatant, the resins were subjected to one or two washing steps, which represented conditions of different selection stringency.

**Figure 4:**
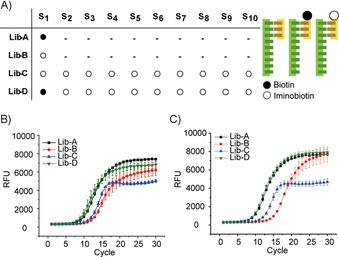
A) Libraries schematic: ten sequences (Table S1) were annealed with a 20‐mer (B′), which was modified with biotin (filled circles), iminobiotin (empty circles), or was unmodified. The selected mixture on SA beads after B) one and C) two washing steps was eluted and subjected to RT‐PCR for diversity evaluation.

As shown in Figure 4 B, after one washing step, more DNA in Lib‐D bound to SA resin than that in the other libraries because all DNAs were modified with either strong or weak binders. Interestingly, its end‐point is lower than that of Lib‐A, which indicates selection with Lib‐D results in a mixture of compounds, although one compound (DNA–biotin) is the major component. For Lib‐C, the curve clearly shows that it contains many binders with similar abundance. As expected, the DNA in Lib‐B is least enriched on SA‐resin. The slightly low end‐point signal could be caused by the unspecific binding of unmodified DNA.

After the second washing step (Figure 4 C), Lib‐A and Lib‐D showed that only one major component was bound to the resin. It is important to note that the assay does not exclude the presence of weak binders, only reflecting their low abundance relative to the potent binder. After two washing steps, Lib‐C remained as a mixture of many different compounds. For Lib‐B, removing unspecific binding of unmodified DNA increased the end‐point value to that of Lib‐A, whereas the increase in amplification cycles is caused by the weak affinity of iminobiotin to SA compared with that of biotin. Although the four libraries are simple models, they are representative of different scenarios in a DECL selection. Lib‐A and Lib‐C are the ideal and most unfortunate cases, respectively. Lib‐B and Lib‐D are more common and improvements to the selection condition are often needed.

In summary, we presented the design of a DNA library to model heterogeneous populations. The RT‐PCR assay can be tuned to an “analytical function”, with different compositions of DNA as inputs, and their diversity and concentrations as outputs. The mentioned DNA libraries were designed as models to verify hypotheses. For example, heterogeneity in a population has shown a protective effect against enzymatic digestion. Likewise, they can also lead to new discoveries. An unexpected reoccurrence of fluorescence caused by the formation of complex DNA structures has been observed if the libraries were subjected to extended PCR cycles. The resulting amorphous 3D structure produced from a highly diverse library (high‐entropy structure) is fundamentally different from that of the low‐entropy structures generated by DNA‐origami technology through designing thousands of complementary DNA strands. The RT‐PCR method also has applications in biochemical analysis, for example, to monitor selection experiments by using DECL^19^, ^20^ (Figure 4). Herein, we have investigated different diversities within the code length of 20 nt. In the future, it will be very interesting to study the minimal code length that allows for efficient diversity evaluation through the RT‐PCR based technique, to achieve cost‐efficient code design for DECLs.

Although the method resembles that of DNA‐based computations with respect to parallel computing,^21^ it functions as an analogue computer with RT‐PCR curves as outputs. Compared with some of the most complex designs of DNA computing, with tens to hundreds of unique DNA strands,^22^ this process can work with datasets of extraordinarily large size, such as the X_20_ library, which contains more than one trillion sequences. By building models of synthetic societies of unprecedented size, this method will open up new venues to study complex and diverse populations.

## Conflict of interest

*F.V.R and Y.Z. are cofounders and shareholders of DyNAbind GmbH*.

## Supporting information

As a service to our authors and readers, this journal provides supporting information supplied by the authors. Such materials are peer reviewed and may be re‐organized for online delivery, but are not copy‐edited or typeset. Technical support issues arising from supporting information (other than missing files) should be addressed to the authors.

Supplementary
