# Supplementary material for: Using a PCR‐Based Method To Analyze and Model Large, Heterogeneous Populations of DNA
Source: Chembiochem. 2020 Jan 7;21(8):1144–9. doi: 10.1002/cbic.201900603 (PMC7217214; doi:10.1002/cbic.201900603)
Supplement: Supplementary file 1 — Supplementary [file CBIC-21-1144-s001.pdf]

CHEMBIOCHEM

Supporting Information

Using a PCR-Based Method To Analyze and Model Large, Heterogeneous Populations of DNA**

Helena Andrade,[a] Alvin K. Thomas,[a] Weilin Lin,[a] Francesco V. Reddavide,[b] and Yixin Zhang*[a]

cbic_201900603_sm_miscellaneous_information.pdf

Table of Contents

1. Experimental Procedures
2. Supporting Information: Figures and Table
3. References
4. Author Contributions

1. Experimental Procedures

Reagents and oligonucleotides. All reagents were purchased from Thermo Fisher Scientific (Germany), unless stated otherwise. All oligonucleotides were purchased from IBA (Germany). Water was nuclease free.

Annealing. The oligonucleotides were diluted in an annealing buffer solution (10 mM Tris-HCl pH 7.5, 100 mM NaCl, 1 mM EDTA) and heated for 5 min at 95 °C. Then, they were slowly cooled to room temperature. When indicated, the annealed products were incubated with 1X SYBR Green I (Lonza, Switzerland).

Polymerase chain reaction (PCR). The PCR amplification was carried out in 50 µL in a peqSTAR2x thermocycler (PeqLab, Germany) using the TrueStart™ Taq DNA polymerase (2 U) system, with 1.5 mM $MgCl_2$, 150 nM primers, 0.2 mM dNTP mix and 100 pM template. The temperature protocol was: 10 min at 95 °C; 5 or 25 amplification cycles of 15 s at 95 °C, 30 s at 60 °C, and 20 s at 72 °C; 30 s at 72 °C; and 10 s at 20 °C.

Real-time PCR (RT-PCR). The RT-PCR experiments were performed using the PerfeCTa SYBR Green SuperMix, which contains AccuStart™Taq DNA polymerase. The amplification reaction was carried out on a PikoReal™ Real-Time PCR System (Thermo Fisher Scientific, Germany), with white 96-well Piko PCR plates and sealed with the respective optical adhesive films. Each reaction well had 10 µL, with 150 nM primers and 100 pM template. The temperature protocol was: 10 min at 95 °C; 20, 30 or 45 amplification cycles of 15 s at 95 °C, 30 s at 60 °C, and 20 s at 72 °C (data acquisition point); 30 s at 72 °C; and 10 s at 20 °C. The results were collected using the PikoReal™ Software 2.2.

Restriction enzymes. The PCR products were diluted: ten times for the five cycles of amplification sample and 100 times for the 25 cycles of amplification sample. Then, 10 µL of this dilution were incubated with 0.5 µL of a restriction enzyme, SmaI (10 U/µL) and/or an isoschizomer of EcoRV (10 U/µL), for 30 min at 30 °C, 37 min at 30 °C, and 20 min at 80 °C. After, 1 µL of the digested sample was added to the RT-PCR mix (described above) and the amplification was monitored for 30 cycles.

Denaturing urea polyacrylamide gel electrophoresis (Urea-PAGE). A 15% Urea-PAGE (TBE-Urea Gel) was pre-ran in 1X TBE buffer, at 160 V and 10 mA, for 30 min. The RT-PCR samples were added 2X TBE Urea Sample Buffer (final volume of 10 µL) and heated to 70 °C for 3 min; then, immediately placed on ice. The gel ran for 1 h, at 160 V and 10 mA. After the run, the gel was stained for 20 min with 1X SYBR Green II (Lonza, Switzerland) in 1X TBE, and read at 470 nm.

Atomic force microscopy (AFM) topographic imaging. AFM topographic images of the RT-PCR products were deposited on freshly cleaved mica in the presence of the imaging buffer (300 mM spermidine trihydrochloride, 300 mM NaCl, 20 mM tris(hydroxymethyl)aminomethane in analytical grade water[1]). Optimal dilutions were deposited on the mica (Plano, Germany) and kept still for 90 s. The substrate was then rinsed with analytical grade water and dried with a steady flow of nitrogen[2]. The images were obtained on air, by contact mode, using a Nanowizard II AFM (JPK, Germany). Silicon nitride cantilevers of 200 µm, with a resonant frequency of about 17 kHz and spring constant of about 0.08 N/m with a gold coating were used. Second order polynomial function was used to remove background slope and the images are shown as a heat map of the surface's topography. DNA origami constructs were imaged as controls to the imaging process.

Library selection: Streptavidin. This selection is based on the high affinity binding model for biotin/iminobiotin and SA[3,4]. The DNA libraries (Figure 4a and Supporting Information, Table S1) were annealed to a final concentration of 100 nM. Then, 100 µL of 10 nM DNA libraries were incubated with 10 µL of SA beads (GE Healthcare, UK), at room temperature for 1 h in a shaker, in the selection buffer (150 mM NaCl, 25 mM $NaHCO_3$, 0.005 % Tween 20, pH 9.2). The SA beads were previously washed three times with the selection buffer and the beads slurry was resuspended in selection buffer before being used. After 1 h, the suspension was centrifuged (5 min, 1000 rpm, 4 °C) and the supernatant discarded. Following washing steps, one or two, were performed. The slurry was then subjected to alkaline denaturation (150 nM NaOH, 3 min at room temperature). Immediately after, the suspension was acidified with 1.5 M acetic acid and again centrifuged. One-µL from the resulting supernatant was added to the RT-PCR mix (described above) and the amplification was monitored for 30 cycles.

2. Supporting Information: Figures and Table

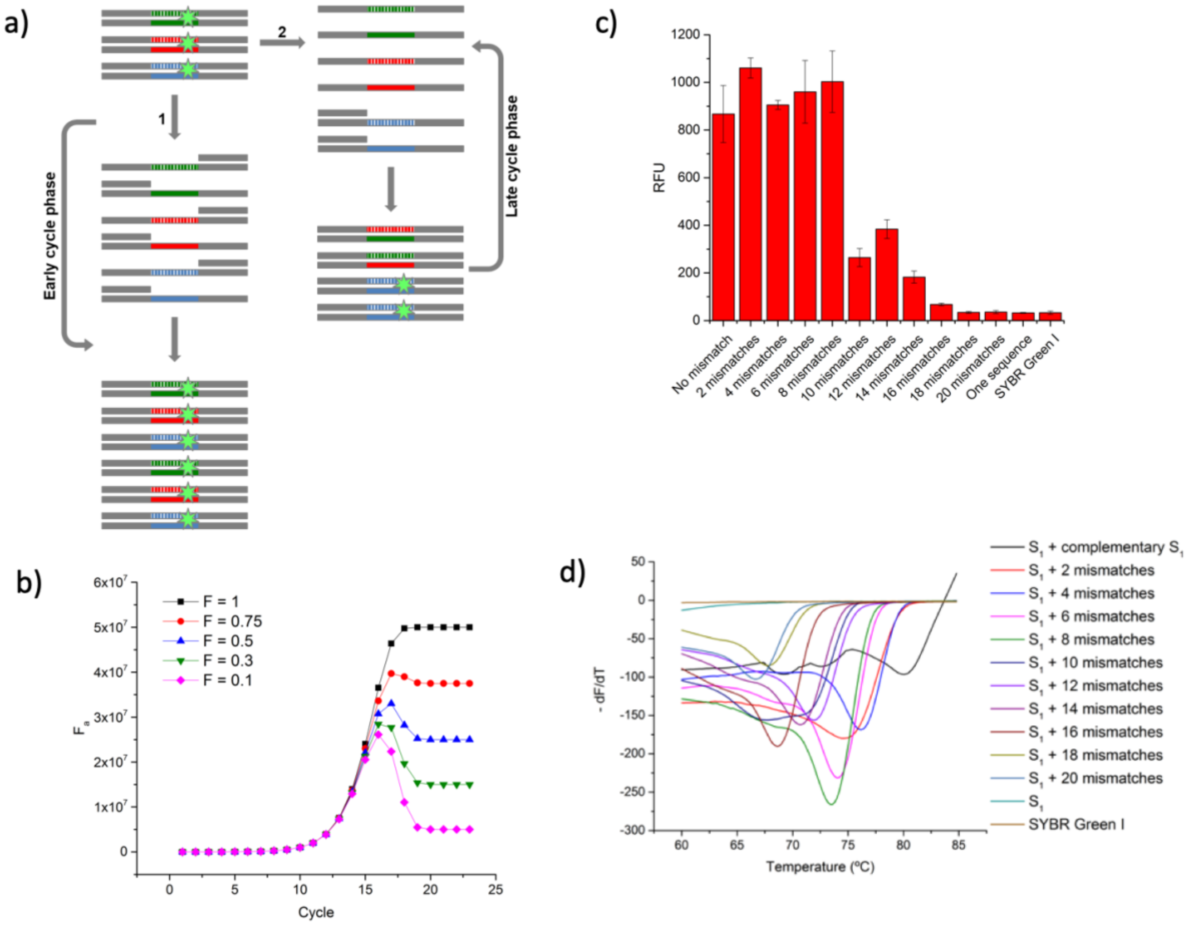

Figure S1. (a) Assay principle. The formation of fully complimentary duplex in the initial PCR cycles (1) and mismatched duplex in the late PCR cycles (2). The mismatched duplex possesses lower F factor and lower affinity to fluorescence dye (green star) at high temperature (e.g., 72 ºC of elongation temperature). (b) Simulation of PCR process using DNA samples of different diversity. (c) Mismatch effect on the fluorescence signal of 100 nM annealed products at 72 ºC of elongation temperature. (d) Melting curves for the mismatched libraries.

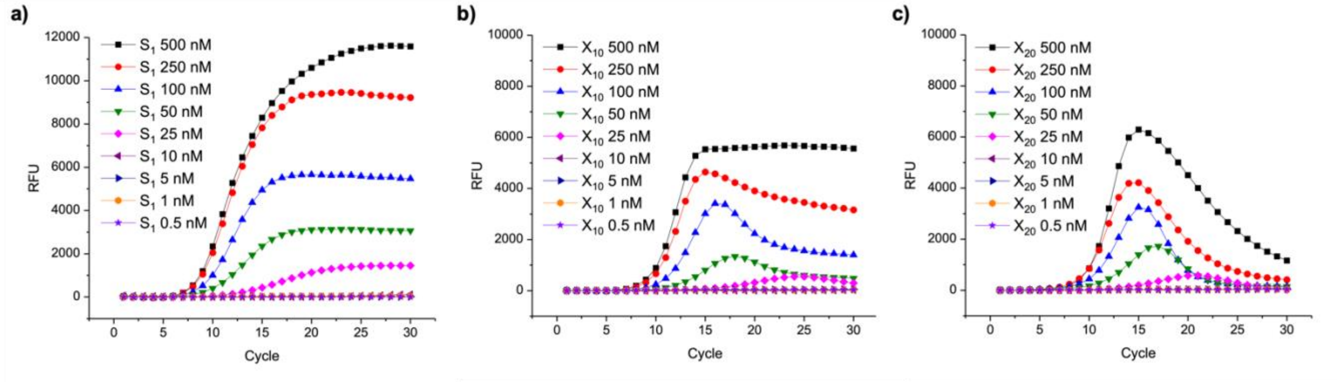

Figure S2. Forward (A) and reverse (B') primers titration for S_1 (a), X_{10} (b), and X_{20} (c), libraries, with final concentrations ranging from 0.5 to 500 nM for both.

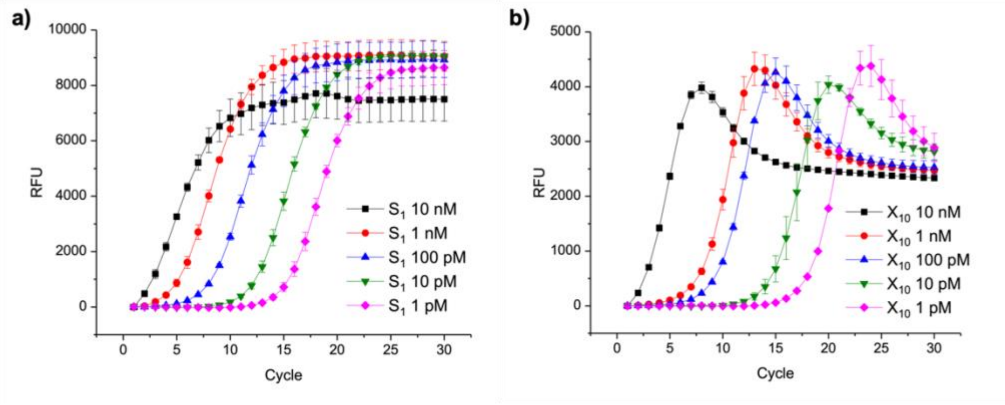

Figure S3. Library titration from 1 pM to 10 nM for S_1 (a) and X_{10} (b).

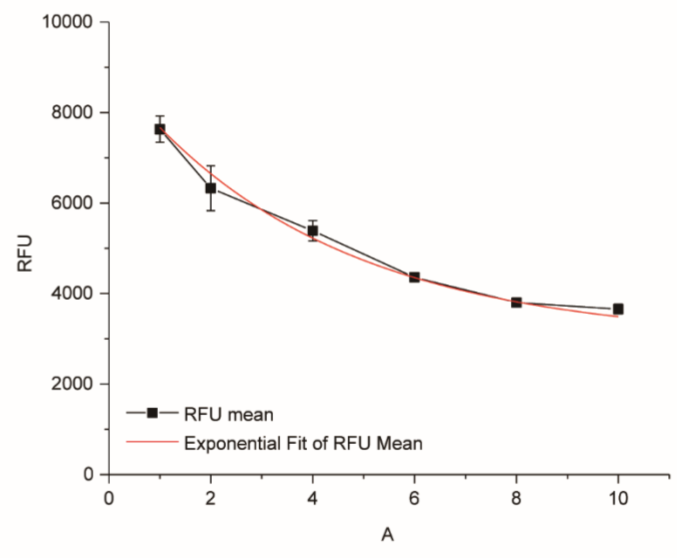

Model	Exponential		
Equation	y = y0 + A*exp(R0*x)		
Reduced Chi-Sqr	1.32116		
Adj. R-Square	0.98763		
		Value	Standard Error
RFU	y0	2971.89722	174.42949
	A	6001.00385	414.90458
	R0	-0.24495	0.02877

Figure S4. Fluorescence (RFU) at 30 cycles of amplification plotted versus the number of high diversity sequences present in the synthetic mixture. The data was extracted from the graph represented on Figure 2d. The resulting curve was then fitted as an exponential function.

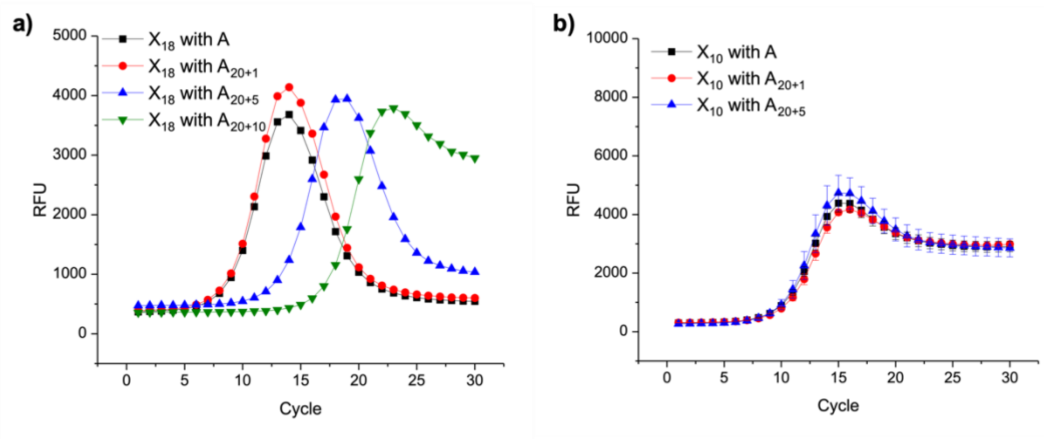

Figure S5. Biased population growth using the longer A primers A_{20+1}, A_{20+5}, or A_{20+10} with the libraries X_{18} (a) and X_{10} (b).

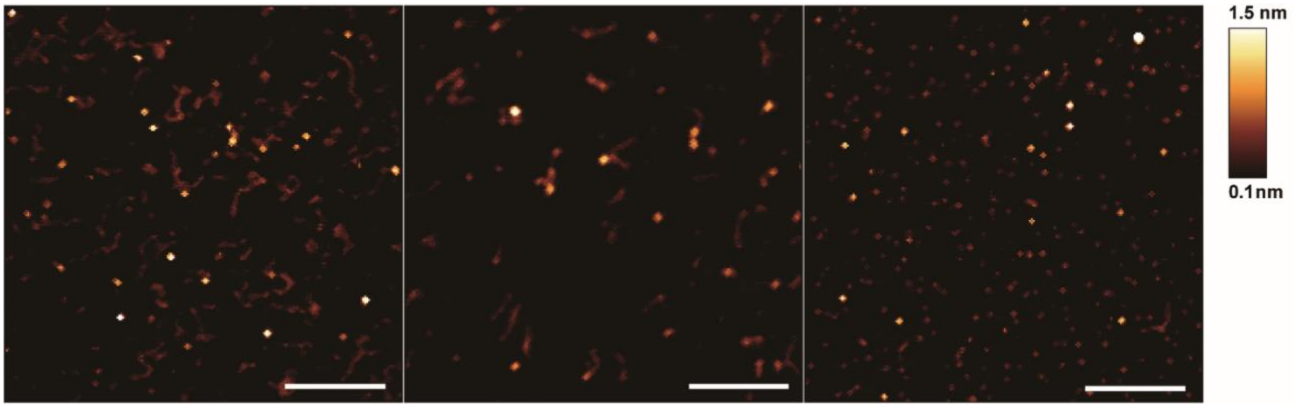

Figure S6. AFM topographic images of PCR products of X_{12} (left) and X_{14} (middle) after 45 cycles, and X_{20} after 20 cycles of amplification (right). Images are 1 µm × 1 µm and the scale bar is 250 nm.

Table S1. DNA sequences.

**Aptamer Libraries**	
**X_{20}**	GACAATTCACACACGTCCGCNNNNNNNNNNNNNNNNNNNNATGAGATCGGAAGAGCGTCG
**X_{18}**	GACAATTCACACACGTCCGCANNNNNNNNNNNNNNNNNNGATGAGATCGGAAGAGCGTCG
**X_{16}**	GACAATTCACACACGTCCGCATNNNNNNNNNNNNNNNNCGATGAGATCGGAAGAGCGTCG
**X_{14}**	GACAATTCACACACGTCCGCATTNNNNNNNNNNNNNNCCGATGAGATCGGAAGAGCGTCG
**X_{12}**	GACAATTCACACACGTCCGCATTCNNNNNNNNNNNNTCCGATGAGATCGGAAGAGCGTCG
**X_{10}**	GACAATTCACACACGTCCGCATTCGNNNNNNNNNNTTCCGATGAGATCGGAAGAGCGTCG
**X_{8}**	GACAATTCACACACGTCCGCATTCGTNNNNNNNNGTTCCGATGAGATCGGAAGAGCGTCG
**X_{6}**	GACAATTCACACACGTCCGCATTCGTANNNNNNAGTTCCGATGAGATCGGAAGAGCGTCG
**X_{4}**	GACAATTCACACACGTCCGCATTCGTAGNNNNCAGTTCCGATGAGATCGGAAGAGCGTCG
**X_{2}**	GACAATTCACACACGTCCGCATTCGTAGGNNTCAGTTCCGATGAGATCGGAAGAGCGTCG
**High Diversity Libraries**	
**S_{1}**	GACAATTCACACACGTCCGCAGTCTGACTGATCACTGGACATGAGATCGGAAGAGCGTCG
**S_{1}'**	CGACGCTCTTCCGATCTCATGTCCAGTGATCAGTCAGACTGCGGACGTGTGTGAATTGTC
**S_{2}**	GACAATTCACACACGTCCGCATCTATGCCTGTATTAAAGCATGAGATCGGAAGAGCGTCG
**S_{3}**	GACAATTCACACACGTCCGCACAAGGGGTCAAGCTCCTGTATGAGATCGGAAGAGCGTCG
**S_{4}**	GACAATTCACACACGTCCGCCCCTCCCTAAACTTTGCCTAATGAGATCGGAAGAGCGTCG
**S_{5}**	GACAATTCACACACGTCCGCGGGATCAGCCTGGGGATACAATGAGATCGGAAGAGCGTCG
**S_{6}**	GACAATTCACACACGTCCGCTATAAATACTACAAGCTCATATGAGATCGGAAGAGCGTCG
**S_{7}**	GACAATTCACACACGTCCGCGGGGCACCGCCAACAGAGATATGAGATCGGAAGAGCGTCG
**S_{8}**	GACAATTCACACACGTCCGCCCGGATTATATCCCATGAGGATGAGATCGGAAGAGCGTCG
**S_{9}**	GACAATTCACACACGTCCGCCTAGTGAAGTGATTCGTTCGATGAGATCGGAAGAGCGTCG
**S_{10}**	GACAATTCACACACGTCCGCGAACGTTAAGGGTGACCGTCATGAGATCGGAAGAGCGTCG
**S_{1} Mismatched Complementary Templates**	
**$S_{1}'_{M2}$**	CGACGCTCTTCCGATCTCATGTCCAGTGAGTAGTCAGACTGCGGACGTGTGTGAATTGTC
**$S_{1}'_{M4}$**	CGACGCTCTTCCGATCTCATGTCCAGTGCGTGGTCAGACTGCGGACGTGTGTGAATTGTC
**$S_{1}'_{M6}$**	CGACGCTCTTCCGATCTCATGTCCAGTACGTGTTCAGACTGCGGACGTGTGTGAATTGTC
**$S_{1}'_{M8}$**	CGACGCTCTTCCGATCTCATGTCCAGCACGTGTGCAGACTGCGGACGTGTGTGAATTGTC
**$S_{1}'_{M10}$**	CGACGCTCTTCCGATCTCATGTCCAACACGTGTGTAGACTGCGGACGTGTGTGAATTGTC
**$S_{1}'_{M12}$**	CGACGCTCTTCCGATCTCATGTCCGACACGTGTGTCGACTGCGGACGTGTGTGAATTGTC
**$S_{1}'_{M14}$**	CGACGCTCTTCCGATCTCATGTCTGACACGTGTGTCAACTGCGGACGTGTGTGAATTGTC
**$S_{1}'_{M16}$**	CGACGCTCTTCCGATCTCATGTATGACACGTGTGTCACCTGCGGACGTGTGTGAATTGTC
**$S_{1}'_{M18}$**	CGACGCTCTTCCGATCTCATGCATGACACGTGTGTCACATGCGGACGTGTGTGAATTGTC
**$S_{1}'_{M20}$**	CGACGCTCTTCCGATCTCATTCATGACACGTGTGTCACAGGCGGACGTGTGTGAATTGTC
**Randomized Regions Libraries**	
**$X_{2/10}$**	GACAATTCACACACGTCCGCNGNCNGNCNGNTNANTNGNCATGAGATCGGAAGAGCGTCG
**$X_{4/5}$**	GACAATTCACACACGTCCGCAGNNTGNNTGNNCANNGGNNATGAGATCGGAAGAGCGTCG
**$X_{10/2}$**	GACAATTCACACACGTCCGCNNNNNGACTGNNNNNTGGACATGAGATCGGAAGAGCGTCG

Restriction Enzymes Libraries	
**X_{EcoRV}**	GACAATTCACACACGTCCGCNNNNNNNGATATCNNNNNNNATGAGATCGGAAGAGCGTCG
**X_{SmaI}**	GACAATTCACACACGTCCGCNNNNNNNCCCGGGNNNNNNNATGAGATCGGAAGAGCGTCG
**Primers**	
**A**	GACAATTCACACACGTCCGC
**A_{18}**	GACAATTCACACACGTCC
**A_{15}**	GACAATTCACACACG
**A_{12}**	GACAATTCACAC
**A_{20+1}**	GACAATTCACACACGTCCGCA
**A_{20+5}**	GACAATTCACACACGTCCGCATTCG
**A_{20+10}**	GACAATTCACACACGTCCGCATTCGTAGGT
**B'**	CGACGCTCTTCCGATCTCAT

3. References

[1] L. Hamon, D. Pastré, P. Dupaigne, C. Le Breton, E. Le Cam, O. Piétrement, *Nucleic Acids Res.* **2007**, *35*, 1–7.

[2] A. Mikheikin, A. Olsen, K. Leslie, B. Mishra, J. K. Gimzewski, J. Reed, *Anal. Chem.* **2014**, *86*, 6180–3.

[3] P. C. Weber, D. H. Ohlendorf, J. J. Wendoloski, F. R. Salemme, *Science (80-.).* **1989**, *243*, 85–8.

[4] S. Melkko, C. E. Dumelin, J. Scheuermann, D. Neri, *Chem. Biol.* **2006**, *13*, 225–31.

4. Author Contributions

H.A. and Y.Z. conceived the project, designed the methods, and experiments; H.A. performed the experiments; H.A. and Y.Z. wrote the manuscript; and A.K.T. performed the AFM topographic imaging; W.L. and H.A. designed and performed the sequencing; F.V.R. assisted with the library preparation.
